# Elicitor from *Trichothecium roseum* Activates the Disease Resistance of Salicylic Acid, Jasmonic Acid, and Ca^2+^-Dependent Pathways in Potato Tubers

**DOI:** 10.3390/jof11070467

**Published:** 2025-06-20

**Authors:** Di Wang, Rong Liu, Haijue Zhang, Zhifei Pei, Xiaoyan Yu, Xueyan Ren, Qingjun Kong

**Affiliations:** Xi’an Key Laboratory of Characteristic Fruit Storage and Preservation, Shaanxi Engineering Laboratory of Food Green Processing and Safety Control, College of Food Engineering and Nutritional Science, Shaanxi Normal University, Xi’an 710119, Chinazhanghaijue@snnu.edu.cn (H.Z.); 18602380017@163.com (Z.P.); xiaoyanyu1988@163.com (X.Y.)

**Keywords:** fungal elicitor, salicylic acid pathway, jasmonic acid pathway, Ca^2+^-dependent, potato

## Abstract

The effects of a fungal elicitor from *Trichothecium roseum* on signal pathways of salicylic acid (SA), jasmonic acid (JA), and Ca^2+^ in potato tubers were investigated. The results showed that fungal elicitor treatment effectively inhibited the lesion diameter of *Fusarium sulphureum* in vivo, which was 17.5% lower than that of the control. In addition, fungal elicitor treatment triggered an increase in O_2_^−^ production and H_2_O_2_ content. The fungal elicitor enhanced the activities and gene expression levels of isochorismate synthase (ICS), phenylalanine ammonia lyase (PAL), allene oxide cyclase (AOC), allene oxide synthase (AOS), lipoxygenase (LOX), and Ca^2+^-ATPase. Furthermore, the fungal elicitor promoted an increase in calmodulin (CaM) content. Protective enzymes (dismutase (SOD), catalase (CAT), polyphenol oxidase (PPO), chitinase (CHI), and β-1,3-glucanase (Glu)) and disease-resistance-related genes (*PR1*, *PR2*, and *PDF1.2*) were induced to be upregulated by elicitor treatment. These results indicated that the fungal elicitor induced disease resistance by accelerating the accumulation of reactive oxygen species (ROS), activating SA, JA, and Ca^2+^ signaling, and upregulating resistance genes. The results of this study revealed the molecular mechanism of fungal elicitor-induced resistance in the potato, which provides a theoretical basis for the mining of new, safe, and efficient elicitor-sourced antifungal agents and is of great importance for the effective control of potato dry rot disease.

## 1. Introduction

The potato (*Solanum tuberosum* L.) is a critical economic crop in China, and it is susceptible to pathogens during postharvest storage, potentially leading to spoilage and causing a great loss [1]. The common diseases affecting potato tubers include late blight, dry rot, soft rot, bud rot, ring rot, black heart rot, and hollow rot, with the principal one being dry rot caused by *Fusarium* spp. [2]. The toxins generated by *Fusarium* spp. include trichothecene mycotoxins, fumonisin mycotoxins, zearalenone, and moniliformin, which would harm people’s health and cause a series of diseases, for instance, acute poisoning, cancer, and abnormalities [3]. To solve these problems, antibacterial and antifungal agents are widely used to preserve potatoes and for their antibacterial activities [2]. However, serious side effects are caused by fungicides, including drug resistance, pollution of the environment, and risks to food safety [4]. Therefore, it is essential to find an environmentally friendly and effective way to keep potato tubers from decaying and extend their shelf life.

Elicitors are a class of substances that can induce defensive responses to abiotic and biotic stresses in plants. Elicitors can induce the expression of disease-resistant genes and resistance proteins, thereby triggering defensive responses against pathogens in plants [5]. Elicitors are divided into biological and non-biological elicitors according to their source. Biological elicitors are divided into endogenous elicitors derived from plants and exogenous elicitors derived from pathogenic or non-pathogenic microorganisms, including polysaccharides, glycoproteins, polypeptides, proteins, oligosaccharides, etc. Non-biological elicitors mainly refer to some non-biological compounds or physical stimulation [6]. In recent years, fungal protein elicitors have attracted more and more attention for their regulation of stresses in plants [7,8]. Some protein elicitors have been separated from *Magnaporthe oryzae* [9], *Alternaria tenuissima* [10], *Botrytis cinerea* [11], and other fungal species, and can regulate signal transduction, activate the immune system, and thereby enhance plant defenses [9].

During the entire life cycle of growth and development, plants are vulnerable to the attacks of various pathogenic agents, such as viruses, bacteria, fungi, oomycetes, etc. Through long-term co-evolution with a variety of pathogenic agents, plants have evolved a diverse and sophisticated immune system. The innate immune system of plants consists of two different levels of responses: pathogen-associated molecular patterns (PAMPs)-triggered immunity (PTI) and effector-triggered immunity (ETI) [12]. The activation of PTI and/or ETI will trigger a series of immune responses, such as a burst of reactive oxygen species, a large influx of Ca^2+^, the activation of receptor-like cytoplasmic kinases (RLCKs), the mitogen-activated protein kinase (MAPK) cascade, and the transcriptional reprogramming of immune-related genes (including plant hormones, pathogenesis-related proteins, etc.) [13,14,15].

A burst of reactive oxygen species (ROS) is one of the primary immune responses of plants against pathogens. It not only directly inhibits the growth of pathogens but also prevents pathogen infection through cell wall thickening and can induce systemic acquired resistance (SAR) in plants [16]. However, excessive ROS production causes cellular oxidative damage to plant cells, leading to the acceleration of cellular senescence. To prevent this, the ROS scavenging system in plants is activated in response to oxidative stress, which includes antioxidant substances (ascorbic acid (AsA), glutathione (GSH), polyphenols, and flavonoids) and antioxidant enzymes (superoxide dismutase (SOD), catalase (CAT), ascorbate peroxidase (APX), and polyphenol oxidase (PPO)) [17]. The antioxidative effects of fungal elicitors have been reported in the grapefruit [18], tomato [19], and broccoli leaf [5]. ROS metabolism is currently receiving attention as a potentially effective target for fungal disease in horticultural products; however, there are few studies thus far on fungal elicitor-mediated ROS production and its interaction with other signaling molecules.

Jasmonic acid (JA) and salicylic acid (SA) are two of the most crucial defense hormones in plants for coping with biotic stresses. JA and SA can activate the signal transduction pathways of JA and SA, as well as the expression of defense genes. They also promote the synthesis or release of resistant metabolites, enzymes, volatile compounds, and other substances within plants, thereby enhancing the plants’ resistance [20,21]. SA is a phenolic compound in plants. Besides participating in the regulation of normal plant physiological activities as a hormone, SA can also act as a key signaling molecule during the plant immune process, triggering the plants to develop systemic resistance against pathogenic agents. JA, a derivative of fatty acids, serves as an endogenous signaling molecule in plants. JA not only regulates plant growth and development but also participates in the response to various biotic and abiotic stresses by inducing the expression of defense genes and the synthesis of resistant substances. The disease-resistant effects of JA and SA have been confirmed in numerous plant species, such as tomatoes, bananas, and cotton. Furthermore, research indicates that the exogenous application of JA and/or SA can also induce an increase in the activity of defense-related substances, such as peroxidase (POD), in various plants, including *Arabidopsis thaliana* [22], rice [23], winter wheat [24], peach [25], and kiwifruit [26]. This enhancement contributes to improving the plants’ resistance to biotic stress.

Acting as a widespread signaling molecule within cells, Ca^2+^ is typically bound to calmodulin (CaM) and activates kinase proteins in response to abiotic and biotic stress, regulating physiological metabolism and gene expression in plants [27]. Studies have shown that Ca^2+^ is closely related to plant disease resistance. Song et al. [28] found that exogenous Ca^2+^ treatment could induce an increase in endogenous Ca^2+^ concentrations in blueberry leaves, enhancing resistance to *Botrytis cinerea*. In tomatoes, elevated Ca^2+^ levels were found to stimulate the accumulation of phytochemicals and activate phenylalaninammo-nialyase (PAL) [29]. It has also been reported that Ca^2+^ might enhance resistance to *Botryosphaeria dothidea* by increasing antioxidant capacity in pear fruit [30].

Numerous reports have centered on the pathogenicity of *Trichothecium roseum* towards fruits and vegetables, while its potential positive applications have been given far less attention [31]. Although previous studies have shown that elicitors can induce disease resistance in various plants, the impact of an elicitor from *T. roseum* specifically on induced resistance has remained poorly understood in potato tubers. In our previous studies, a fungal elicitor named elicitor was isolated and purified from *T. roseum*, which was proven to be able to initially activate phenylaprapanoid metabolism and induce disease resistance in potatoes [32]. However, its detailed regulatory mechanism is unclear. With this in mind, the current study aimed to investigate changes in the signaling pathways of salicylic acid (SA), jasmonic acid (JA), and Ca^2+^ in order to illustrate the regulatory mechanisms underlying induced resistance.

## 2. Materials and Methods

### 2.1. Chemicals and Reagents

Potato dextrose agar (PDA) and potato dextrose broth (PDB) were obtained from Solarbio Chemical Co. (Beijing, China). All other reagents were of analytical grade and bought from Beijing Chemical Corporation (Beijing, China).

### 2.2. Collection, Treatment, and Storage of Potato Tubers, Pathogens, and the Fungal Elicitor 

*Fusarium sulphureum* was obtained from the Institute of Plant Protection, Gansu Academy of Agricultural Sciences, China. The *F. sulphureum* was cultured on potato dextrose agar (PDA) for seven days, and the spore suspension (10^6^ spores × 10^−3^ L^−1^) was prepared for inoculation.

The identification, isolation, and purification of fungal elicitors were performed according to the method of Yu et al. [32]. The mycelia of *T. roseum* were collected after seven days of growth in potato dextrose broth (PDB) at 25 °C and dried with a suction filter device. The mycelia were extracted with Tris-HCl buffer (pH 7.0) to prepare the elicitor. After centrifugation at 12,000× *g* for 30 min and purification with an ammonium sulfate precipitation, the fungal elicitor was obtained. The protein concentration was measured as described in Bradford [33].

Potato tubers (*Solanum tuberosum* cv. Xindaping) at optimum maturity (firmness of 50 N ± 1 N, total soluble solid content of 5.8% ± 6.6%, and starch content of 13.9% ± 0.3%) were collected from a market (Dingxi city, Gansu province, China) and transported to the laboratory for immediate treatment. The potato tubers collected were of the same size and without surface blemishes. The tubers were washed and dipped into sodium hypochlorite before treatment. After washing with sterile water, the potato tuber was air-dried. During the storage period, potato samples were stored at 5 °C.

Based on our previous study [32], potato tubers were cut into slices (35 mm in diameter and 10 mm in thickness). Then, 20, 40, 60, and 80 μg mL^−1^ of elicitor solution were applied to the surface of the potato slices, and sterile water was used as the control. Agar discs containing *F. sulphureum* mycelium were taken with a punch and inoculated in the center of the potato slices. All inoculated slices were incubated in the dark at 25 °C for 0, 2, 4, 8, 12, 24, 48, and 72 h, and then 3.0 g of tissue (3 mm in thickness) was collected. Each treatment consisted of 360 slices (3 replicates of 120 each). The tissue samples were promptly frozen in liquid nitrogen and stored at −80 °C for in-depth analysis.

### 2.3. In Vivo Inhibitory Activity Assessment of the Elicitor

*F. sulphureum* was cultured on potato dextrose agar (PDA) for seven days, and the spore suspension (10^6^ spores × 10^−3^ L^−1^) was prepared for inoculation. In total, 300 potatoes were selected and randomly divided into two groups of 150 (control group and elicitor-treated group), with 3 replicates of 50 each. The potato’s surface was sterilized with 75% alcohol and air-dried, and then the surface of the tubers was punched uniformly with a perforator (4 mm in diameter). The holes were inoculated with 20 μL of the spore suspension, dried, and stored at 22 (±2 °C). Potato samples in the elicitor-treated group were sprayed with 20, 40, 60, and 80 μg mL^−1^ of the elicitor at room temperature, and the control group was sprayed with sterile water. Wounded and inoculated potatoes were stored at 22 °C, and the diameters of the dry rot lesions were determined using the criss-cross method after 16 d.

### 2.4. The Rate of Superoxide Anion (O_2_·^−^) Production and Hydrogen Peroxide (H_2_O_2_) Content Assay

The H_2_O_2_ content was determined using an H_2_O_2_ assay kit (Nanjing Jiancheng Bioengineering Research Institute, Nanjing, China) following the manufacturer’s instructions. The H_2_O_2_ content was expressed based on the fresh weight as μmol g^−1^.

The production of O_2_·^−^ was determined by inhibition and using the superoxide anion assay kit (Nanjing Jiancheng Bioengineering Research Institute, Nanjing, China), following the manufacturer’s instructions. The production rate of O_2_·^−^ was expressed based on the fresh weight as μmol g^−1^ min^−1^.

### 2.5. Detection of SA Content and Activities of Isochorismate Synthase (ICS) and PAL

The salicylic acid content was determined following the method of Pál et al. [34], with slight modifications. Frozen samples (0.1 g) were weighed and crushed with liquid nitrogen. A total of 20 mL of 90% methanol was added, vortexed for 1 min, and then centrifuged at 12,000× *g* for 30 min to collect the supernatant. The supernatant was collected by centrifugation at 12,000× *g* for 30 min after vortexing for 1 min. The supernatant was combined, dried with a rotary evaporator, and dissolved in 15 mL of 5% trichloroacetic acid. Then, it was extracted by adding 40 mL of a mixture of ethyl acetate and cyclohexane (1:1, *v*/*v*). The extraction was repeated once and the organic phases were combined, transferred to a rotary evaporator, dried, fixed with 10 mL (0.2 M, pH 5.5) sodium acetate, and stored at 4 °C after filtration through a 0.45 μm filter membrane. It was determined by high-performance liquid chromatography. The chromatographic parameters were as follows: column: Waters HSS T3 (50 × 2.1 mm, 1.8 μm); mobile phases: A: methanol; B: sodium acetate (6:4, *v*/*v*; pH 5.5); detection wavelength: 320 nm; flow rate: 0.8 mL min^−1^; column temperature: 30 °C; injection volume: 20 μL.

The ICS assay was performed according to the method of Poulsen et al. [35], with slight modifications. Frozen samples were completely crushed under liquid nitrogen, mixed with the ICS enzyme extract (0.1 M Tris-HCl at pH 7.5, 10% glycerol, 1 mM DTT, 0.2 mM PMSF, 1 mM EDTA), and centrifuged at 13,000× *g* for 20 min at −4 °C. The supernatant was taken as 125 μL, and the same volume of the reaction solution was added and mixed well (0.1 mM pH 7.5 Tris-HCl, 1 mM chorismate, 10 mM MgCl_2_). This was kept at 30 °C for 1 h and the reaction was completed with the reaction solution. At the end of the reaction, 62.5 μL of termination solution (methanol:sec-butanol [1:1, *v*/*v*]) was added to the sample tube. In the control tube, the enzyme solution was replaced by the termination solution and kept at 30 °C for 1 h. Finally, 100 μL of the reaction mixture was mixed with 1.4 mL of NaH_2_PO_4_ buffer (pH 7.0), and the fluorescence detector (FLD) values were determined using a HITACHI F-2700 fluorescence spectrophotometer (the excitation wavelength was 302 nm and the emission wavelength was 412 nm). The mixture was boiled for 15 min and cooled to room temperature. The FLD value was measured again, and the difference between the FLD values before and after heating was used to calculate the ICS enzyme activity.

PAL activity was measured by a PAL test kit from Nanjing Jiancheng Bioengineering Research Institute, Nanjing, China), following the instructions of the test kit. PAL activity was expressed based on the fresh weight as U g^−1^.

### 2.6. Detection of JA Content and Activities of Allene Oxide Cyclase (AOC), Allene Oxide Synthase (AOS), and 13-Lipoxygenase (LOX)

The jasmonic acid content was determined using the method of Li et al. [36], with slight modifications. Frozen samples (0.1 g) were crushed with liquid nitrogen. The supernatant was then centrifuged at 13,000× *g* for 10 min at 4 °C for 10 min. The supernatant was purified through a C-18 column and stored in a refrigerator at 4 °C for the determination of jasmonic acid using high-performance liquid chromatography (HPLC). The separation was performed on a Waters HSS T3 column (50 × 2.1 mm, 1.8 μm) with the mobile phases A: ultrapure water (containing 0.1% acetic acid) and B: acetonitrile (containing 0.1% acetic acid), and a gradient elution (0–15 min, 15%→35% A; 15–40 min, 35%→80% A) at a flow rate of 0.3 mL/min and a column temperature of 40 °C. The injection volume was 2 μL. The jasmonic acid content was expressed based on the fresh weight as mg g^−1^.

AOC activity was determined using an AOC ELISA kit (Camilo Bioengineering Co., Ltd., Nanjing, China), following the instructions of the test kit. AOC activity was expressed based on the fresh weight as U g^−1^.

AOS activity was determined using an AOS ELISA kit (Camilo Bioengineering Co., Ltd., Nanjing, China), following the instructions of the test kit. AOS activity was expressed based on the fresh weight as U g^−1^.

LOX activity was measured using a LOX Assay Kit test kit (Nanjing Jiancheng Bioengineering Research Institute, Nanjing, China), following the instructions of the test kit. PAL activity was expressed based on the fresh weight as U g^−1^.

### 2.7. Detection of Calmodulin (CaM) Content and Ca^2+^-ATPase Activity

CaM content was determined using a CaM ELISA kit (Shanghai Sailing Biological Institute, Shanghai, China), following the instructions of the test kit. CaM content was expressed based on the fresh weight as ng g^−1^.

Ca^2+^-ATPase activity was measured using a Ca^2+^-ATPase test kit (Nanjing Jiancheng Bioengineering Research Institute, Nanjing, China), following the instructions of the test kit. Ca^2+^-ATPase activity was expressed based on the protein level as μmol Pi mg^−1^ port h^−1^.

### 2.8. Detection of SOD, CAT, PPO, Chitinase (CHI), and β-1,3-Glucanase (Glu) Activities

SOD activity was determined using a SOD test kit (Nanjing Jiancheng Bioengineering Research Institute, Nanjing, China), following the instructions of the test kit. SOD activity was expressed based on the fresh weight as U g^−1^.

CAT activity was determined using a CAT test kit (Nanjing Jiancheng Bioengineering Research Institute, Nanjing, China), following the instructions of the test kit. CAT activity was expressed based on the fresh weight as U g^−1^.

PPO activity was determined using a PPO test kit (Nanjing Jiancheng Bioengineering Research Institute, Nanjing, China), following the instructions of the test kit. PPO activity was expressed based on the fresh weight as U g^−1^.

CHI activity was determined using a CHI test kit (Nanjing Jiancheng Bioengineering Research Institute, Nanjing, China), following the instructions of the test kit. CHI activity was expressed based on the fresh weight as U g^−1^.

β-1,3-glucanase activity was determined using a β-1,3-glucanase test kit (Beijing Solaibao Technology Co., Ltd., Beijing, China), following the instructions of the test kit. β-1,3-glucanase activity was expressed based on the fresh weight as U g^−1^.

The Coomassie Brilliant Blue method was used to quantify the protein content [33].

### 2.9. Gene Expression Analysis Using Quantitative Real-Time PCR (qRT-PCR)

Total RNA was isolated from ground tissues using the cetyltrimethylammonium bromide (CTAB) method and extracted using a Takara RNA extraction kit (Takara Biotechnology, Shiga, Japan). First-strand cDNA was synthesized using a first-strand cDNA Synthesis Kit (Sangon Biotech, Shanghai, China).

The corresponding *F. sulphureum* nucleotide sequences were obtained from the GENBANK database and used to design gene-specific primer pairs by employing Primer 5.0 software (Table 1). qRT-PCR was performed to measure the expression levels of *F. sulphureum* genes. The SYBR Green PCR Premix Ex Taq ™ (Takara Biomedicals, Shiga, Japan), cDNA, forward and reverse primers, and ROX reference dye II were added to an ABI 7000 instrument (Applied Biosystems, Foster City, CA, USA) for the reaction. The operation was as follows: at 95 °C for 10 s, at 95 °C for 5 s with 40 cycles, and at 60 °C for 40 s. The *F. sulphureum* actin gene (StActin, X55751.1) was the internal reference. Relative quantifications were then calculated using the 2^−∆∆CT^ method, and the CT values from the *F. sulphureum* actin gene were used to normalize all qRT-PCR reactions.

### 2.10. Statistical Analysis

Each treatment included three biological replicate samples, and the SPSS11.0 software package (SPSS Inc., Chicago, IL, USA) was used to analyze the data. Data were subjected to one-way analysis of variance (ANOVA) and Duncan’s post hoc test, where significance was set at a *p*-value < 0.05. The resulting data are presented using the mean ± standard deviation.

## 3. Results

### 3.1. Effect of the Elicitor on Spore Germination and the Lesion Diameters of Dry Rot Disease of Potato Tubers In Vivo

**Figure 1:**
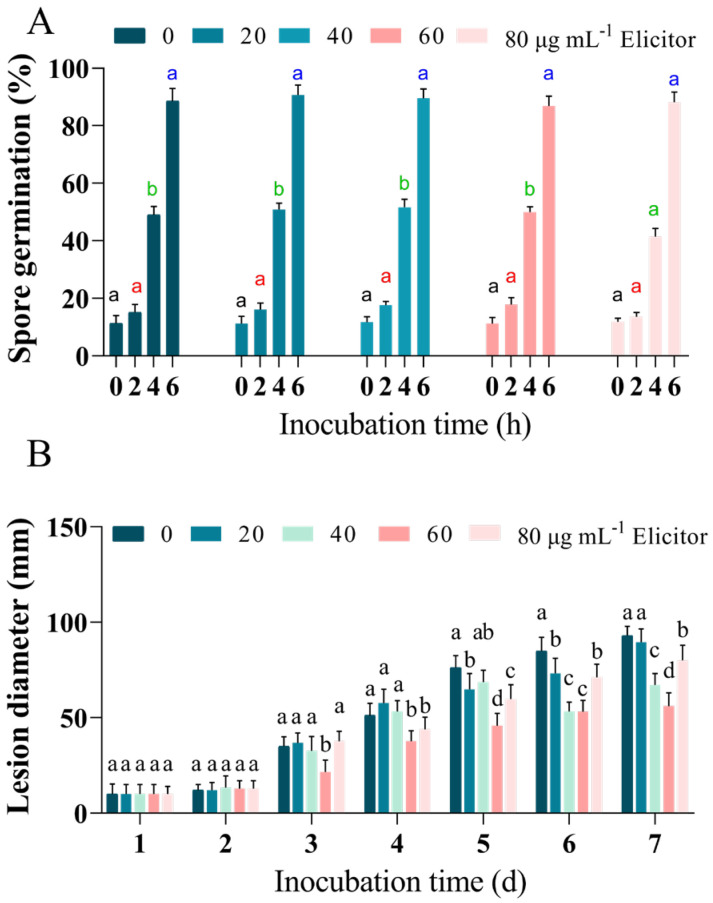
Effects of different concentrations of inducers on the dry rot spot diameters of potato tubers with in vivo spore germination (0–6 h), and the in vitro mycelial growth (0–7 d) of *F. sulphureum*; (**A**) spore germination; (**B**) mycelial growth; values are expressed as mean ± SD. Different letters indicate significant differences (*p* < 0.05).

All concentrations of elicitor treatments did not significantly inhibit *F. sulphureum* spore germination compared to the control (*p* < 0.05) (Figure 1A). The potato tuber inoculation result showed that the 20 μg mL^−1^ and 40 μg mL^−1^ elicitor treatments did not significantly inhibit or promote the mycelial growth of *F. sulphureum*, and 60 μg mL^−1^ and 80 μg mL^−1^ elicitor treatments significantly inhibited the growth at the late stage (*p* < 0.05). On the 7th day, 60 μg mL^−1^ and 80 μg mL^−1^ elicitor treatments significantly inhibited the growth of *F. sulphureum* mycelia in the group, which decreased by 39.78% and 13.98%, respectively, compared with the control group at day 7 (Figure 1B).

### 3.2. Effect of the Elicitor on the Generation of O_2_·^−^ and the Content of H_2_O_2_

**Figure 2:**
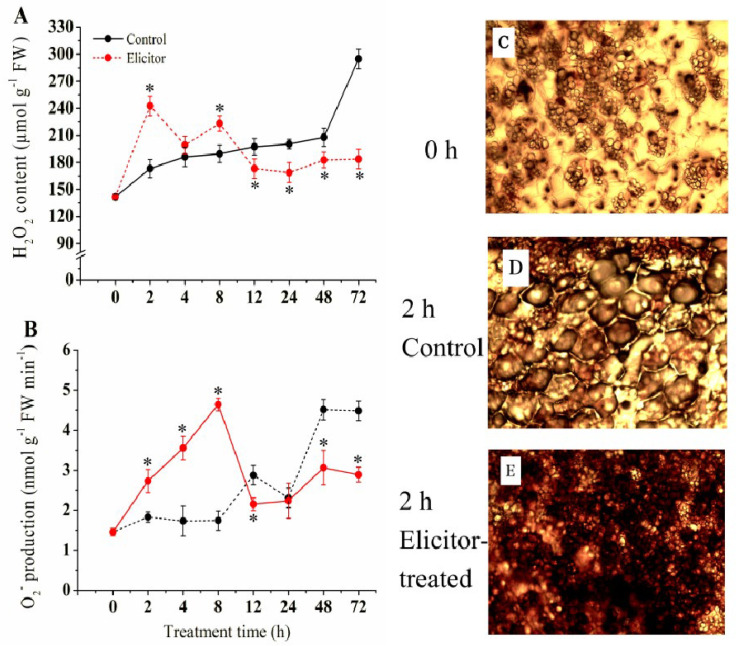
Effect of the elicitor on H_2_O_2_ content (**A**), the production rate of O_2_·^−^ (**B**), and H_2_O_2_ DAB staining observations at 0 h (**C**), after 2 h of treatment in the control group (**D**), and after 2 h of treatment in the elicitor-treated group (**E**) of potato tubers. Scale bars: 50 μm (**C**–**E**). Values are presented as means ± SD. Asterisks (*) indicate significant differences in the figure (*p* < 0.05).

As shown in Figure 2A, the content of H_2_O_2_ in all groups increased in the early stages. The H_2_O_2_ content in the elicitor group peaked at 2 h and 8 h, which were 23.3% and 19.1% higher than the control, respectively. Afterward, the content of H_2_O_2_ decreased, which was lower than that of the control group at 48 h. The H_2_O_2_ content of the control group reached a maximum at 72 h, which was 1.6 times that of the elicitor group. The production of O_2_·^−^ of the elicitor group increased significantly compared to the control group in the first 24 h of storage. The O_2_·^−^ production of the elicitor group peaked at 8 h, which was 2.27 times that of the control group (Figure 2B). The elicitor-treated and control groups both showed peaks at 8 and 48 h, respectively, which then declined. H_2_O_2_ accumulation in tubers was observed using DAB staining. As can be seen from the figure, compared to 0 h, 2 h after exciton treatment, a large amount of H_2_O_2_ had accumulated in the potato tubers of the treatment group compared to the control group (Figure 2C–E).

### 3.3. Effect of the Elicitor on SA Synthesis

**Figure 3:**
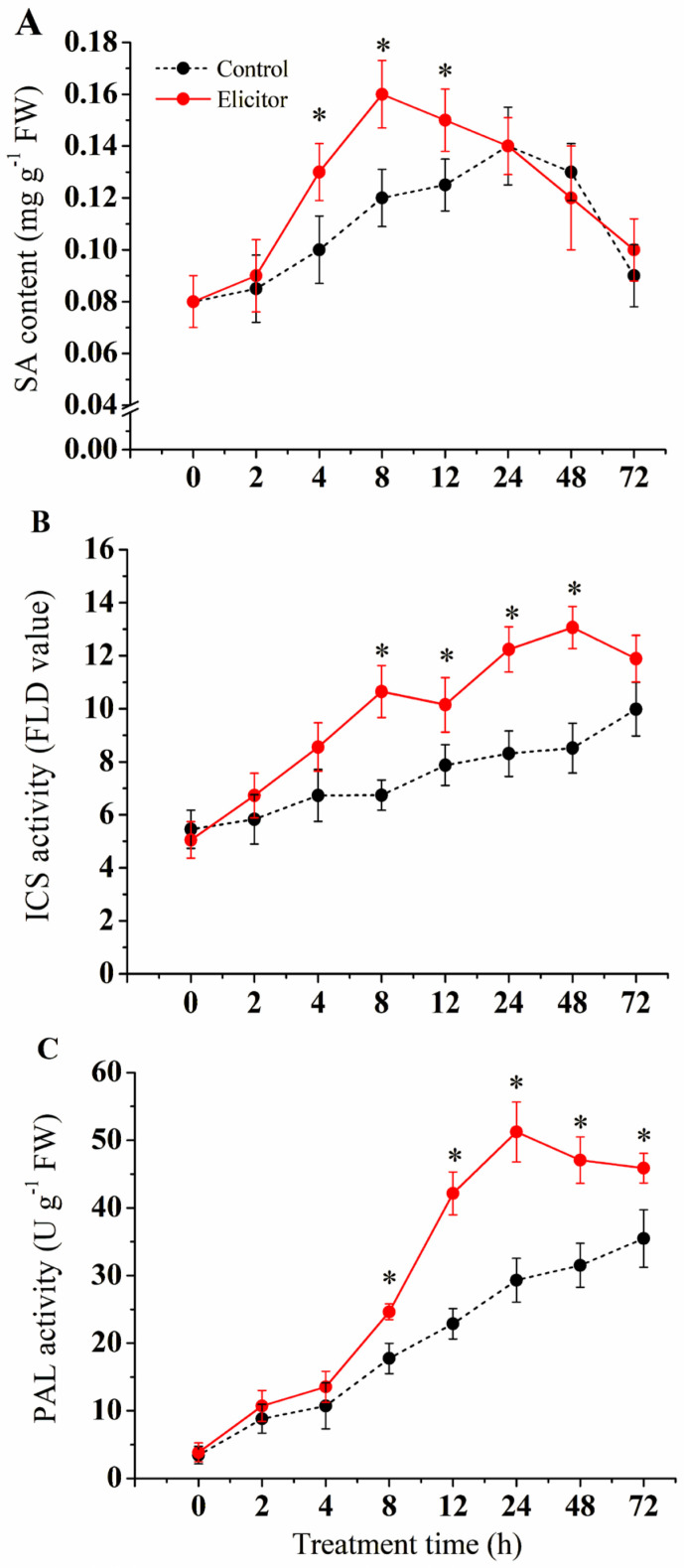
Effect of the elicitor on SA content (**A**) and the activities of ICS (**B**) and PAL (**C**) in potato tubers. Values are presented as means ± SD. Asterisks (*) indicate significant differences in the figure (*p* < 0.05).

As shown in Figure 3, elicitor treatment significantly induced the synthesis and accumulation of SA in the potatoes (*p* < 0.05). The SA content gradually increased, reaching a peak at 8 h, and then gradually decreased, with the SA content in the elicitor-treated group being 33.3% higher than that of the control group at 8 h (Figure 3A). ICS and PAL enzyme activities were activated by elicitor treatment and were significantly higher than those of the control group (*p* < 0.05) from 2 h−72 h. ICS enzyme activity peaked at 8 h and 48 h (Figure 3B). PAL activity peaked at 24 h, when it was 74.8% higher than that of the control group (*p* < 0.05) (Figure 3C).

### 3.4. Effect of the Elicitor on the Expression of Key Genes Related to the SA Signaling Pathway

**Figure 4:**
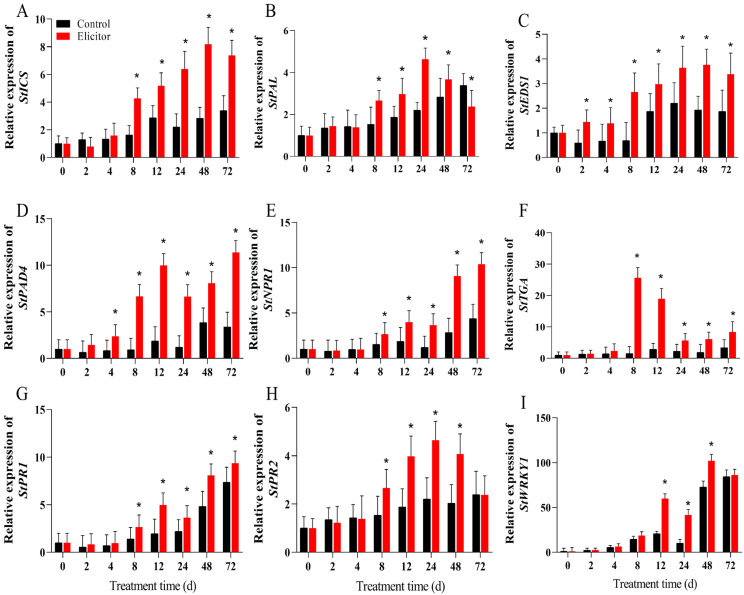
Effect of the elicitor on gene expression levels of *StICS* (**A**), *StPAL* (**B**), *StEDS* 1 (**C**), *StPAD4* (**D**), *StNPR1* (**E**), *StTGA* (**F**), *StPR1* (**G**), *StPR2* (**H**), and *StWRKY1* (**I**) in potato tubers. Values are presented as means ± SD. Asterisks (*) indicate significant differences in the figure (*p* < 0.05).

As shown in Figure 4, the qRT-PCR results indicated that the expression levels of *StICS* and *StPAL*, the key genes for salicylic acid synthesis, were significantly upregulated in the elicitor-treated group from 8 h onwards, and the expression levels of *StICS* and *StPAL* peaked at 48 h and 24 h, at which time they were 2.88 times and 2.10 times of those in the control group, respectively (Figure 4A,B). *Enhanced disease susceptibility 1* (*StEDS1*) (Figure 4C) and *phytoalexin decient 4 (StPAD4)* (Figure 4D), which are located upstream of the signaling pathway that promotes salicylic acid synthesis, were significantly upregulated at the early stages of elicitor treatment, and their expression levels were maintained at high levels from 8h to 72 h after elicitor treatment. In the salicylic acid signaling pathway, the expression levels of the salicylic acid receptor regulator, *non-expressor of pathogenesis-related genes 1* (*StNPR1*) (Figure 4E), the transcription factor *TGACG motif-binding factor* (*StTGA*) (Figure 4F), and the *conserved heptapeptide motif WRKYGQK 1* (*StWRKY1*) (Figure 4G) were upregulated at 8 h after elicitor treatment. The expression levels of *StNPR1* peaked at the end of the treatment period (48 h and 72 h), when they were 3.20 times and 2.37 times higher than that of the control group, respectively. *StTGA* expression levels were significantly upregulated (*p* < 0.05) at 8 h and 12 h, when they were 16.8 times and 6.61 times higher than those of the control group, respectively, and then began to gradually decline. At 48 h, *StWRKYl* (Figure 4G) expression was 1.40 times higher than that of the control group. The expression levels of *pathogenesis-related 1 (StPR1)* and *StPR2* (Figure 4G,H), which are important disease-course-related genes in the salicylic acid signaling pathway, were gradually increased by elicitor treatment at 8 h, and remained at a high expression level for 48 h.

### 3.5. Effect of the Elicitor on JA

**Figure 5:**
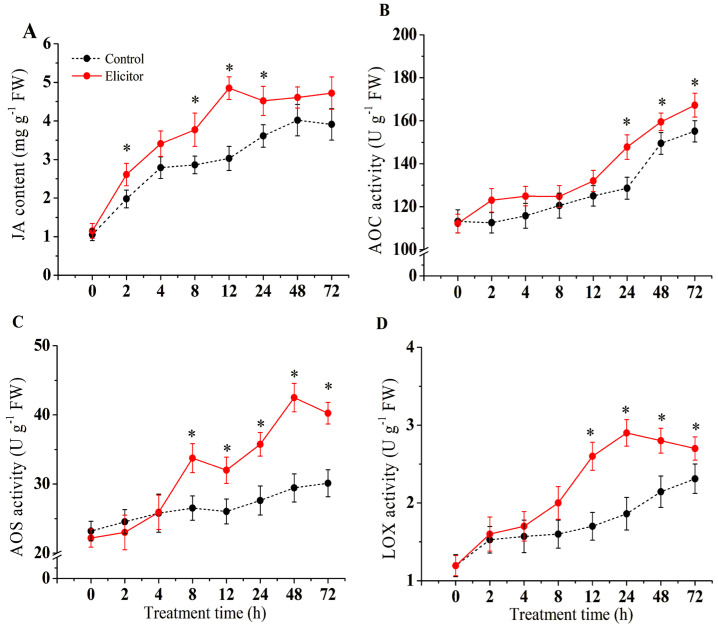
Effect of the elicitor on JA content (**A**) and the activities of AOC (**B**), AOS (**C**), and LOX (**D**) in potato tubers. Values are presented as means ± SD. Asterisks (*) indicate significant differences in the figure (*p* < 0.05).

As shown in Figure 5, elicitor treatment significantly induced the synthesis and accumulation of JA in potatoes (*p* < 0.05). The JA content increased gradually, reaching a peak at 12 h when the JA content of the elicitor-treated group was 60.1% higher than that of the control group (Figure 5A). The activities of the key enzymes for JA synthesis, AOC, AOS, and LOX, were activated by elicitor treatment and were significantly higher than those of the control group at the late stages of treatment (*p* < 0.05) (Figure 5B–D). AOS activity peaked at 8 h and 48 h, when AOS activity was 27.2% and 44.3% higher than that of the control group, respectively (Figure 5C). LOX activity peaked at 24 h, when LOX activity was 55.8% higher than that of the control group (Figure 5D).

### 3.6. Effect of the Elicitor on the Expression of Key Genes Related to the JA Signaling Pathway

**Figure 6:**
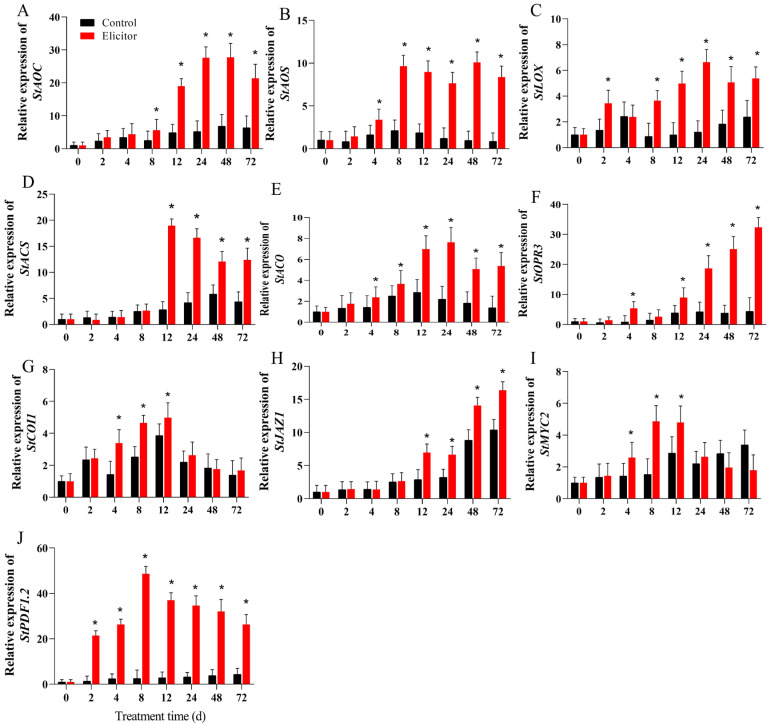
Effect of the elicitor on gene expression levels of *StAOC* (**A**), *StAOS* (**B**), *StLOX* (**C**), *StACS* (**D**), *StACO* (**E**), *StOPR3* (**F**), *StCOI1* (**G**), *StJAZ1* (**H**), *StMYC2* (**I**), and *StPFD1.2* (**J**) in potato tubers. Values are presented as means ± SD. Asterisks (*) indicate significant differences in the figure (*p* < 0.05).

As shown in Figure 4, the qRT-PCR results indicated that the expression levels of the key enzymes of jasmonic acid synthesis, the *StAOS* and *StLOX* genes, were significantly upregulated in the elicitor-treated group from 8 h (Figure 6B,C), and the expression level of the *StAOC* gene was significantly upregulated in the elicitor-treated group from 12 h (Figure 6A). At 24 h, the expression levels of *StAOS*, *StLOX*, and *StAOC* in the elicitor-treated group were 6.33 times, 5.50 times, and 5.31 times higher than those of the control group at 24 h, respectively. The expression levels of *StOPR3*, the key gene for JA synthesis, and the key regulator of the pathway, *jasmonate ZIM-domain 1* (*StJAZ1*), gradually increased from 12 h and peaked at 72 h (Figure 6F,H). The expression levels of the JA pathway regulator *Stcoronatin insensitive 1 (COI1)* and the transcription factor *myelocytomatosis 2* (*StMYC2*) tended to increase and then decrease, peaking at 12 h, at which time the expression levels of *StCOI1* and *StMYC2* in the elicitor-treated group were 1.28 times and 1.67 times higher than those in the control group, respectively (Figure 6G,I). In addition, the JA pathway-mediated defense gene *plant defensin 1.2* (*StPDF1.2*) was significantly upregulated by the elicitor, and its expression level remained high from 2 h to 72 h.

### 3.7. Effect of the Elicitor on the Calcium Signaling Pathway

**Figure 7:**
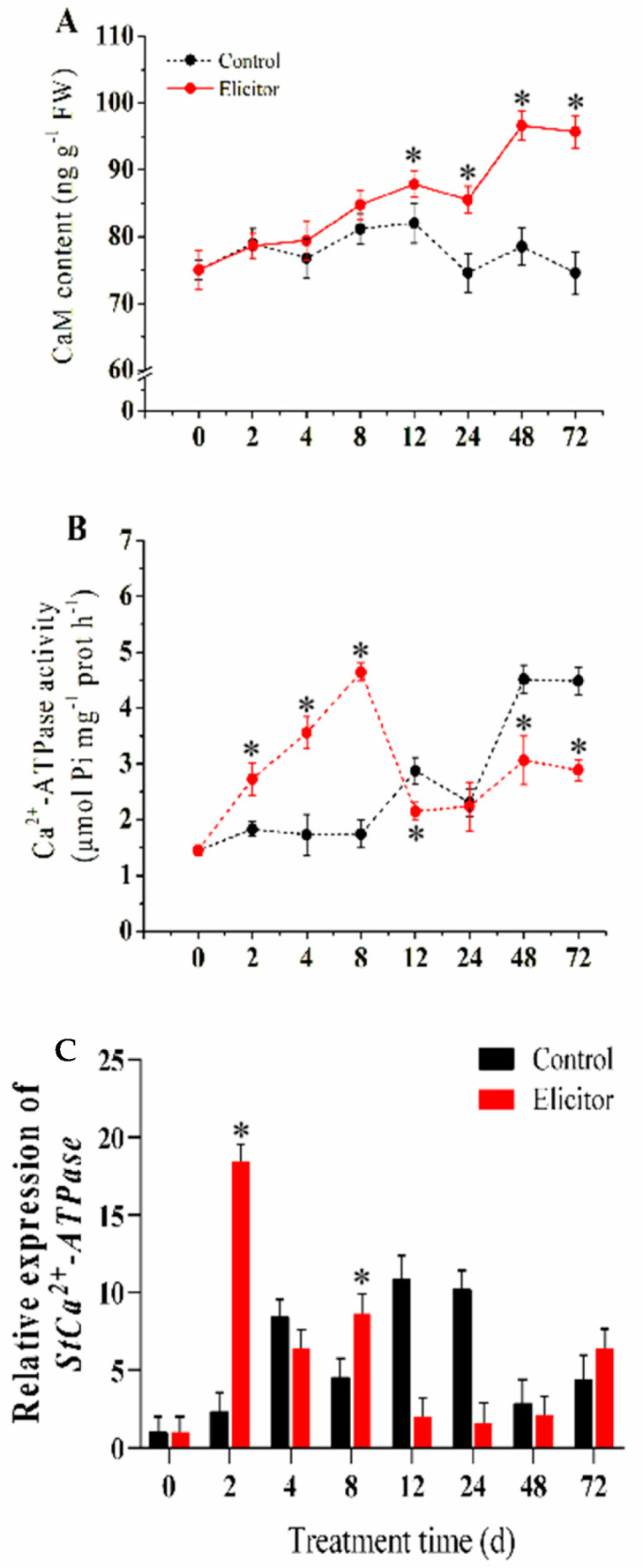
Effect of the elicitor on CaM content (**A**), Ca^2+^-ATPase activity (**B**), and the expression level of *StCa^2+^-ATPase* (**C**) in potato tubers. Values are presented as means ± SD. Asterisks (*) indicate significant differences in the figure (*p* < 0.05).

The elicitor treatments induced CaM accumulation in potato tubers throughout the whole storage period. The CaM content reached a peak at 12 h and 48 h, respectively, when the activity was 1.07 times and 1.23 times higher than in the control group (Figure 7A). The Ca^2+^-ATPase activity of the elicitor group increased and peaked at 8 h, when it was 2.67 times higher than in the control group (Figure 7B). The qRT-PCR results showed that the expression level of *StCa^2+^-ATPase* in the elicitor group significantly increased at 2 h and 8 h after treatment, when it was 7.84 times and 19.1 times that of the control group, respectively (Figure 7C).

### 3.8. Effect of the Elicitor on the Activities of Defense-Related Enzymes

**Figure 8:**
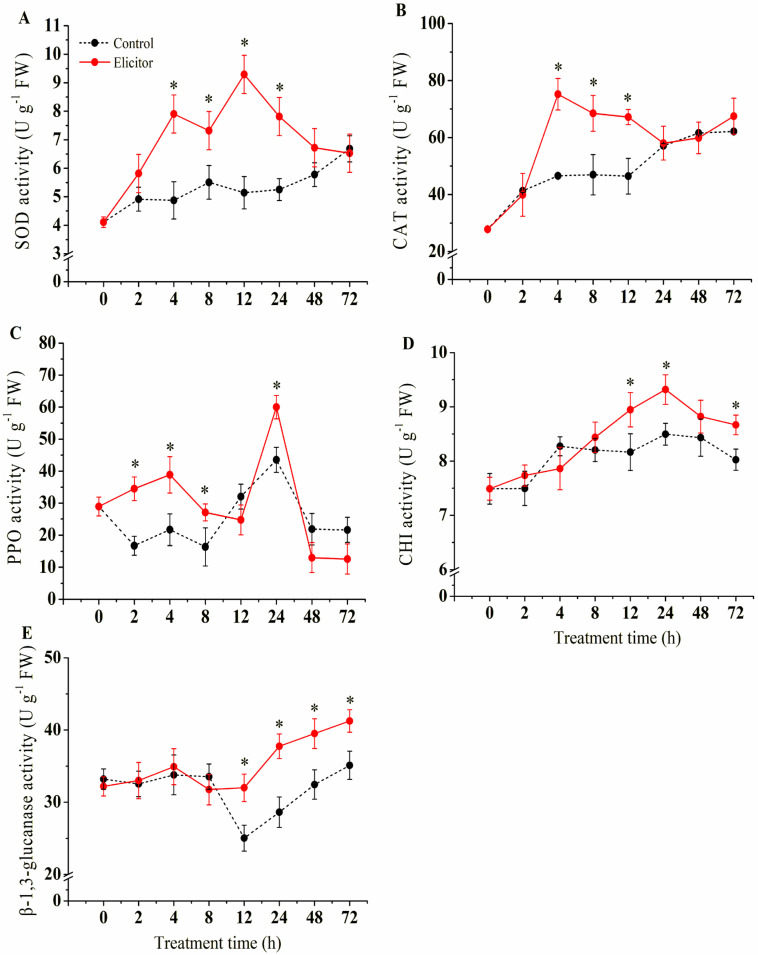
Effect of elicitor on activities of SOD (**A**), CAT (**B**), PPO (**C**), CHI (**D**) and Glu (**E**) of potato tuber. Values are presented as means ± SD. Asterisks (*) indicate significant differences in the figure (*p* < 0.05).

As shown in Figure 8A, the SOD activity of the elicitor group increased and peaked at 12 h, when it was 1.81 times that of the control group. The change in the CAT activity of the elicitor groups was increased in the early stages and then decreased (Figure 8B). The CAT activities of the elicitor group reached their maximum at 4 h, when they were 1.61 times those of the control group. Compared to the control group, PPO activities increased in the elicitor group (Figure 8C). The PPO activities peaked at 24 h in the elicitor-treated group before declining and being maintained at a stable active level (Figure 8C). As shown, the elicitor treatment induced an increase in CHI activity, which reached its maximum at 24 h, when it was 1.10 times higher than that of the control group during the same period (Figure 8D). Elicitor treatment resulted in an increase in β-1,3-glucanase activity at later stages, which was 31.9% higher than the controls at the end of treatment (Figure 8E).

### 3.9. Effect of the Elicitor on the Gene Expression Levels of Defense-Related Genes

**Figure 9:**
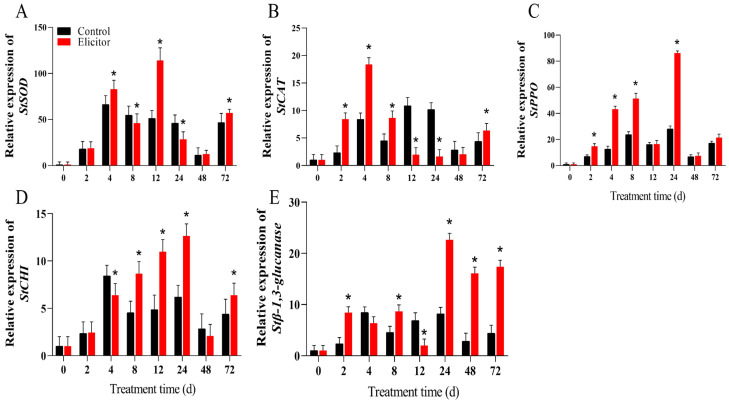
Effect of the elicitor on gene expression levels of *StSOD* (**A**), *StCAT* (**B**), *StPPO* (**C**), *StCHI* (**D**), and *StGluGlu* (**E**) in potato tubers. Values are presented as means ± SD. Asterisks (*) indicate significant differences in the figure (*p* < 0.05).

The expression levels of *StSOD* (Figure 9A) and StCAT (Figure 9B) in the elicitor-treated group increased. The expression levels of *StSOD* in the elicitor groups reached their maximum at 12 h, when they were 2.22 times that of the control group. *StCAT* reached its highest levels at 4 h, when it was 2.18 times that of the control group. The expression levels of *StPPO* and *StCHI* in the elicitor group significantly increased and peaked at 24 h after treatment, when they were 3.07 times and 2.04 times that of the control group, respectively (Figure 9C,D). *StGlu* was significantly upregulated (*p* < 0.05) in the later stages of elicitor treatment, with peak expression levels at 24 h, and remained highly expressed from 24 h to 48 h (Figure 9E).

## 4. Discussion

There have been a large number of studies on whether elicitors could effectively stimulate plant autoimmunity and improve their disease resistance. The use of elicitors to induce disease resistance for the protection of plants has been reported in various horticultural products [37]. At the same time, fungal elicitors would not cause the generation of pathogen resistance as a result of acting on the plant. Hence, some speculated that fungal elicitor products have great potential for bacteriostasis [5]. This study indicated that the elicitor-treated group exhibited significant inhibition effects in potato tubers in vivo and in PDA plate culture media compared to the control group (Figure 1A,B). In tobacco leaves, elicitors from *Alternaria tenuissima* improved resistance to the tobacco mosaic virus (TMV) [10]. Similar effects of fungal elicitors on postharvest products have been reported in apples [38], pears [39], and cucumbers [40], among others.

Fungal infections could induce the massive production and accumulation of ROS in plants, which was also considered one of the earliest responses of host plants to pathogen invasion. Early ROS production could induce multiple resistance responses in the host, such as cell wall thickening, a hypersensitive response (HR), activation of resistance genes, and accumulation of resistant substances [20]. According to the results of this study, treatment with fungal inducer 80 could induce the rapid accumulation of O_2_·^−^ and H_2_O_2_ in potato tubers during the early stages (Figure 2). It has also been reported that elicitors from *B. cinerea* improved disease resistance in *Arabidopsis thaliana* by accelerating ROS accumulation in the early stages [41]. In addition, oxidative burst and nitric dioxide (NO) accumulation have been observed in moss plants [42] and cotton [43], which supported the conclusion that fungal elicitors could induce a rapid increase in H_2_O_2_ and NO production, leading to enhanced resistance against pathogens.

In response to pathogen invasion, plants can precisely adjust their metabolites to defend against pathogen infection and maintain normal physiological states [44]. As defensive secondary metabolites, plant hormones are extensively involved in plant immune responses to pathogenic bacteria, among which the defensive regulatory roles of SA and JA have been well-studied [45]. ICS and PAL are key enzymes in the SA biosynthesis pathway, regulating SA production through distinct synthetic routes [46]. *EDS1* and *PAD4*, located upstream of the SA signaling pathway, amplify SA signals via SA-associated feedback loops to promote SA production [47]. Accumulated SA activates the expression of the downstream receptor regulatory factor *NPR1* and facilitates its binding with *TGA* transcription factors to trigger the expression of pathogenesis-related proteins [48]. Additionally, SA accumulation induces *WRKY1* expression, promoting the transcriptional binding of *WRKY1* with *PR1* and *PR2* to enhance plant disease resistance [49].

Pathogenesis-related proteins (PRs) are a class of proteins induced and expressed in plants upon pathogen infection. The plant PR protein family consists of 17 members, with most PRs exhibiting antifungal activity [50]. *PR1* not only possesses antimicrobial activity but also induces stress resistance in plants, being recognized as a marker of plant disease resistance and systemic acquired resistance [50]. *PR2* belongs to the β-1,3-glucanase family and degrades β-1,3-glucans in fungal cell walls, thereby inhibiting fungal growth and spread [51]. Studies have shown that the salicylic acid signaling pathway induces the expression of *PR2*, aiding plants in establishing long-term immunity against pathogens and playing a crucial role in plant acquired resistance. This study reveals that elicitors extracted from *T. roseum* upregulate the activity and gene expression of key SA biosynthesis enzymes (ICS and PAL) and trigger the expression of upstream SA signaling genes *EDS1* and *PAD4* in the early stages, promoting SA production. SA accumulation further enhances the expression of regulatory factors *NPR1*, *TGA*, and *WRKY1*, subsequently leading to the high-level expression of *PR1* and *PR2* genes. These findings are consistent with previous results where the elicitor AsES, derived from *Acremonium strictum*, upregulated the expression and content of SA pathway genes in *Arabidopsis*, induced high-level expression of *ArPR1*, and promoted systemic resistance of *Arabidopsis* against *Botrytis cinerea* [52].

JA synthesis can be accelerated under biotic and abiotic stress conditions. Its biosynthesis starts with α-linolenic acid (LeA), which is converted to 13-hydroperoxylinolenic acid (13-HPOTE) by LOX. 13-HPOTE is catalyzed by AOS and AOC to produce 12-oxo-phytodienoic acid (OPDA), which is then converted to JA by the action of OPDA reductase (OPR3) to produce JA [53]. JAZ and COI1 are important regulators in the jasmonate pathway. The transcription factor MYC2 is a key regulator in the JA signaling pathway [53]. In strawberry fruit, exogenous methyl jasmonate (JA donor) treatment initiated a defense response mediated by the upregulation of different defense-related genes and was accompanied by the upregulation of MYC2 and JAZ1 [54]. PDF1.2 encodes a plant defensin, which is induced by JA, ethylene, fungal infections, and ROS [52]. It has been shown that JA and ethylene synergistically induce the expression of defense-related genes in a wide range of plants [52]. The present study showed that the elicitor induced and increased the SA content and the activities of LOX, AOS, and AOC in potato tubers (Figure 5). The underlying mechanism is to upregulate the expression of genes related to JA synthesis at the transcriptional level (Figure 6). In addition, the expression of key regulatory genes of the JA pathway, OPR3, COI1, JAZ1, MYC2, and PDF1.2, was upregulated accordingly, which may play a positive role in enhancing resistance in potatoes [55]). Hanan et al. (2020) also found that fungal elicitors activated the expression of LOX, AOS, and AOC, promoted JA accumulation, and enhanced resistance to Myzus persicae in tomato plants [56]. Another study found that an elicitor from *Lecanicillium lecanii* induced upregulation of LOX, AOS, AOC, and PR1 expression and promoted JA accumulation in tomato plants, thereby increasing the resistance of tomatoes to Myzus persicae [57]. It has been shown that elicitors from *Acremonium strictum* induced the upregulation of *PDF1.2*, *AOS*, and *PR1*, thereby enhancing disease resistance to Boytrytis cinerae in Arabidopsis, which is consistent with our results [52]. These results support the notion that elicitor-enhanced pathogen resistance in potato tubers may be due to early SA accumulation and the activation of related defense genes.

Early signaling molecules, including Ca^2+^, NO, and ROS, were induced by elicitors in plants, and these signal molecules interact with downstream signals to activate plant defense responses [58]. Ca^2+^ is crucial for transmembrane transport and signal transduction, and CaM may act as a receptor or a messenger in this process [27]. This study indicated that the elicitor accelerated CaM accumulation and increased the activities of Ca^2+^-ATPase and NADPH oxidases (NOX), resulting in a higher intracellular ROS level in the early stages and improved disease resistance in potato tubers. In particular, Ca^2+^ may be involved in upstream signaling pathways induced by the elicitor. Research on rice demonstrated that Ca^2+^ stimulated defense responses against blast fungus infections, while the blockade of Ca^2+^ signaling (treatment with a Ca^2+^ inhibitor) reduced this effect [59]. Able et al. [60] have come up with similar results. It has been found that disease resistance was positively associated with Ca^2+^ content in tobacco. These findings support our speculations.

Although ROS are essential for enhancing disease resistance in plants, the excessive accumulation of ROS can be detrimental to plant cells, leading to lipid peroxidation and damage to membrane lipids and proteins. The plant antioxidant system consists of antioxidant enzymes such as SOD, CAT, and PPO, which are responsible for scavenging excess ROS and protecting plants from oxidative damage [17]. In the present study, we showed that the fungal elicitor increased the activities of SOD, CAT, and PPO in potato tubers (Figure 8A–C). The underlying mechanism involved upregulating the expression of genes related to oxidation–reduction reactions at the transcriptional level (Figure 9). Gao et al. [61] found that an endophytic fungal elicitor could activate SOD and CAT, thereby improving the plant’s defense response. Another study revealed that an elicitor from *Trichoderma harzianum* increased SOD, CAT, and PPO activity and regulated antioxidant activity in tomato plants, conferring resistance to *Fusarium wilt* disease [19]. These results further support the opinion that elicitor-enhanced pathogen resistance in potato tubers is probably due to early ROS burst induction and the activation of antioxidant defense.

The above results suggest that fungal elicitor treatments improved disease resistance in potato tubers and reduced the impact of disease development by inducing ROS accumulation and activating SA, JA, and Ca^2+^ signaling pathways. Furthermore, it is reasonable to speculate that SA, JA, and CaM accumulation may be associated with ROS production and that these signaling molecules are involved in defense responses and mediate inducer-induced host resistance at an early stage. In addition, whether Ca^2+^ signaling is involved in SA, JA, or ethylene-interacting signaling pathways requires further investigation.

## 5. Conclusions

In conclusion, the present study demonstrated that a fungal elicitor from *T. roseum* can induce systemic resistance against *F. sulphureum* in potato tubers. The growth of *F. sulphureum* was inhibited by increasing SA and JA content and improving ROS levels. In addition, the fungal elicitor induced the activities and gene expression levels of ICS, PAL, AOC, AOS, LOX, and Ca^2+^-ATPase, and maintained high levels of SA and JA in potato tubers. Upregulation of resistance genes (*SOD*, *CAT*, *PPO*, *CHI*, *Glu*, *PR1*, *PR2*, and *PDF1.2*) may also play a positive role in potato defenses against *F. sulphureum*. The above results indicate that SA, JA, and CaM signaling pathways were activated, which might have contributed to the enhanced resistance against *F. sulphureum* in potato tubers. However, considering that disease resistance is likely to be modulated by multiple pathways, further studies should be performed to explore other potential factors and their underlying mechanisms.

## Figures and Tables

**Table 1:** Primer sequences used for real-time quantitative PCR (qRT-PCR).

Gene	Forward Primer (5′-3′)	Reverse Primer (5′-3′)
Actin	CTGGGCAGAAGGAAAAGAGG	AATACTACGCAGGCTCATCAAAC
ICS	TGCTCGCTCTCGTCTTCACCTC	ATCTCTTGAATCGCCTTGGCATCC
PAL	GGTCACTGCCTCGGGTGAT	CCTGCCAGTGAGCAAACCA
EDS1	CTTCTGCACTGGGAATAGGA	TTCGGAACTCAGTTGAGAGG
PAD4	TTGCATTACCTTTGGCTCTC	CATGATGGGGAAAAGAACAG
NPR1	GCACTTGAATCGGCTTAGGG	GCTTCTTCAGTTGACGCTCT
PR1	GGCATCCCGAGCACAAAAT	CTGCACCGGAATCAAGT
PR2	GTGAAGCTGGTTTGGGAAATG	TTGCCAATCAACGTCATGTCTAC
TGA	GGAGTATGGTCAGTGGGTGGA	GCTCAAAGTAGTGGTTCAGGCAA
WRKY1	GAAGAATAAAGCCGGGTTCTTGG	CTTACACGATTTGATCACCTCATCC
AOC	CCCGATCTGCCATCTGAGTT	GCCCCATCCTCACAAGCTT
AOS	TTGAAACCCTAGATAAGGAAATGGC	AAGCCCCCAACGCCGACTTATCAA
LOX	TGGGTGGCTTCTGCTCTT	TTTGGAACTGGGCTGTGA
OPR3	TGATTTCCCCGACTTCAGCT	CATGAGATGCACGACCAACA
JAZ1	TTCATCATCGTCATCGTCGT	GGGGTTTTGTTTGTTGGCTA
COI1	TTGGAGGAGTTTGGTGGTGG	GAGGGAAAACTAGTGCGGCAT
MYC2	AAGAACAAGCGTAGAGCGTCGTC	CACCGCATCATCACCTCCACTTC
PFD1.2	TCTTTTGCCTCGTCCTTGTT	TTGTGACCCCATGGTTTGTA
Ca^2+^-ATPase	CACTATGTTGCCAGCGGACTGC	GCCACCTCAGTTCCAGCGATTC
SOD	CTCCTGAAGATGAGGTGCGT	GAACCACAATAGAAGGGCAGAA
CAT	AGTCGGAGGAGCAAATCACAG	GCAGAGCCACAACAGCATCCC
PPO	TTGCCAATCAACGTCATGTCTAC	TGAACCGGGGTATGAGGGAT
CHI	ATTGGAAACGGATATGCTCCA	TCCTTACCTGAACGCCTGTCA
Glu	TCTTTTCCGCCTTCCTCTG	AACTTCGTTGGGGTTGTCTTT

## Data Availability

The original contributions presented in this study are included in the article. Further inquiries can be directed to the corresponding authors.
